# Characteristics of perinatal depression in rural central, India: a cross-sectional study

**DOI:** 10.1186/s13033-018-0248-5

**Published:** 2018-11-12

**Authors:** Sujit D. Rathod, Simone Honikman, Charlotte Hanlon, Rahul Shidhaye

**Affiliations:** 1 Department of Population Health, London School of Hygiene & Tropical Medicine, London, UK; 2 Perinatal Mental Health Project, Alan J Flisher Centre for Public Mental Health, Department of Psychiatry and Mental Health, University of Cape Town, Cape Town, South Africa; 3 Centre for Global Mental Health, Health Services and Population Research Department, Institute of Psychiatry, Psychology and Neuroscience, King’s College London, London, UK; 4 Department of Psychiatry, School of Medicine, College of Health Sciences, Addis Ababa University, Addis Ababa, Ethiopia; 5 Public Health Foundation of India, New Delhi, India

**Keywords:** Perinatal, Depression, Women, Epidemiology, India

## Abstract

**Background:**

Perinatal depression is associated with negative effects on child behavioural, cognitive and emotional development, birth outcomes, and physical growth. In India, increased priority accorded to mental health programs mean it is now possible to reduce the population-level burden of perinatal depression. In this secondary analysis of two studies, we aimed to describe the epidemiological features of depression among community- and facility-based samples of perinatal women from rural central India, and to describe the help-seeking behaviours from those women who screened positive for depression.

**Methods:**

The Community Study was a multi-round population-based cross-sectional survey (n = 6087). The Facility Study was a multi-round facility-based cross-sectional survey (n = 1577). Both studies were conducted in Sehore District, Madhya Pradesh between 2013 and 2017. Field workers conducted structured interviews with perinatal women. The questionnaire had sections relating to sociodemographic characteristics, depression screening using the Patient’s Health Questionnaire (PHQ9), treatment seeking for depression-related symptoms, and disability. Using data pooled from both studies, we tested each characteristic for association with the total screening score and with screening positive for depression.

**Results:**

We identified 224 perinatal women from the Community Study and 130 perinatal women from the Facility Study, of whom 8.8% and 18.5% screened positive for depression, respectively. For the continuous PHQ9 score, there was evidence of a “U” shaped association with age, and positive associations with pregnancy, disability score, suicidality and being a health facility attendee. For the binary PHQ9 score, there was evidence of positive associations with pregnancy, disability score, suicidality and being a health facility attendee.

**Conclusions:**

This study highlights where the largest population-level variations in perinatal depression symptoms are present in this Indian sample, for which mental health service provision should be made a priority. Epidemiological evidence generated by this study, as well as new evidence on peer-delivered interventions for perinatal depression, must be utilized by policy-makers to prioritize mental health services for mothers along with maternal and child health services.

**Electronic supplementary material:**

The online version of this article (10.1186/s13033-018-0248-5) contains supplementary material, which is available to authorized users.

## Background

Perinatal depression is defined as depression occurring in a woman while she is pregnant or within 12 months of delivery [1]. A global systematic review by Stein et al., found evidence that perinatal depression is associated with negative effects on child behavioural, cognitive and emotional development, and mixed evidence for negative effects on birth outcomes (i.e. pre-term and birth weight) and physical growth [2]. In a subsequent systematic review by Gelaye et al. of perinatal depression among low- and middle-income country (LMIC) populations there was largely consistent evidence of negative impacts for birth outcomes and physical growth [3], though insufficient evidence is available for the other impacts which were identified in the global review.

In LMIC the prevalence of antenatal depression is estimated to be 15–20%, and 20% for postnatal depression [1, 3], though with considerable variation between countries [4–6]. Within India, prevalence estimates for antenatal or postnatal depression range between 6 and 48% [7–15]. The high variation in estimates could be due the use of screening tools; two studies from South India which used diagnostic interviews had prevalence estimates of 11% and 16% [8, 14].

From these India-based studies, a range of risk factors for perinatal depression have emerged in domains relating to sociodemographic and economic status; child gender preference; and family and marital characteristics [7–10, 13, 14, 16]. Identifying women for treatment of depression has the potential to lead to direct health benefits for women and indirect benefits for their children. The need to improve child health is urgent in India, where 30% of newborns are low birth weight [17] and 38% of children under 5 are moderately or severely stunted [18].

In India, launch of the National Mental Health Policy and Mental Healthcare Act and increased priority accorded to mental health programs mean that there is now an opportunity to reduce the population-level burden of perinatal depression. To inform planning of health promotion activities and service delivery, we have characterized community- and facility-based samples of perinatal women from rural central India who were screened for depression, and described their help-seeking behaviour.

## Methods

### Setting

Sehore District, which is located in the central Indian state of Madhya Pradesh, has a population 1.3 million, of whom 81% live in a rural area and 68% are literate [19]. In Madhya Pradesh, maternal health services are readily accessible: 90% of mothers were immunized for tetanus prior to their last birth and 81% of births are in health facilities [20]. In collaboration with the Programme for Improving Mental Health Care (PRIME) research consortium [21], the state government started integrating mental health care services for depression into the primary health care sector, which includes perinatal health services, in Sehore District in 2013 [22]. Starting in 2016, nurses in district hospitals across the state are receiving training to detect perinatal depression and provide basic psychological interventions.

### Community Study

A detailed description of the Community Study is available [23]. Briefly, the Community Study was a multi-round, population-based survey which aimed to estimate the change in treatment coverage among adults (age ≥ 18 years) affected by depression or by alcohol use disorder after implementation of PRIME. In the baseline (pre-implementation) round, research assistants selected 89 villages randomly in Sehore District, selected one voting polling station randomly from each village (range 1–5 stations), and downloaded the list of registered voters for that station from the government election commission website. At the time the study was designed, we assumed that the voter registration list was fairly comprehensive and suitable for use as a sampling frame for the population of potential health service users. After the baseline survey occurred, the implementation area was redrawn, reflecting a decision to focus programme activity on 188 villages in one sub-district. The follow up survey sample included all 188 villages, and all voter lists in each village. In both rounds, from each voting list, the research assistant selected adults randomly to recruit into the study. The baseline survey (n = 3220) was conducted between May 2013 and March 2014, the follow-up survey (n = 2914) was conducted between October and January 2017, and independent samples were drawn in each round.

### Facility Study

The Facility Study aimed to estimate the change in detection of depression and of alcohol use disorders by primary care clinicians working in three public clinics in one sub-district of Sehore District. Research assistants recruited a systematic random sample of adults (age ≥ 18 years) who were exiting their clinical consultations. The baseline (pre-implementation) survey (n = 760) was conducted between August and October 2013, the follow up (post-implementation) survey (n = 817) was conducted between September and October 2016, and independent samples were drawn in each round.

### Data collection and measures

In both studies, Hindi-speaking interviewers used a structured questionnaire application administered on an Android tablet device to ask participants about their sociodemographic characteristics (i.e. age, religion, caste, educational attainment, marital status, housing quality, land ownership, and parity, and age and sex of living children), and health-related status (i.e. pregnancy, depression, disability, and recent suicidal ideation). The response options for housing quality are widely understood in this setting to correspond to homes which are low quality, intermediate quality and high quality.

Interviewers screened participants for depression with the 9-item Patient’s Health Questionnaire (PHQ9) [24], which has been validated in India [25]. Each item relates to a DSM-IV criterion for major depressive disorder and is scored from 0 (“not at all”) to 3 (“nearly every day”) with a recall period of 2 weeks. Four score categories have been established: 0–4 normal, 5–9 mild depressive symptoms, 10–14 moderate depressive symptoms ≥ 15 moderately severe to severe depressive symptoms [26, 27]. We considered a total PHQ9 score of 10 or more to be a positive screen for depressive symptoms. A meta-analysis of PHQ9 validation studies found that this cut off score has 85% sensitivity and 89% specificity for detecting depression [28]. The Cronbach’s alpha for the PHQ9 for this sample was 0.67.

To assess disability status over the past 3 months, interviewers administered the 12-item World Health Organization Disability Assessment Schedule (WHODAS) 2.0 [29], and administered an adaptation of the Composite International Diagnostic Interview (CIDI) suicidality module [30] to assess suicidal ideation in the past 12 months.

In the Community Study, interviewers asked PHQ9-positive participants whether they had sought treatment (i.e. from the specialist, generalist or complementary health provider, and the nature of the treatment). In the Facility Study, interviewers asked PHQ9-positive participants about their clinical consultation (i.e. any diagnoses made, and the nature of any advice, treatment or referral provided).

As part of the recruitment process, interviewers gave adults oral and written information about the study and participating adults affirmed their consent with a signature or thumb print. Participants who affirmatively responded to questionnaire items about suicidality received a referral to a research psychiatrist. The study protocols were approved by Sangath (Goa, India), the University of Cape Town (South Africa) and World Health Organization (Geneva, Switzerland).

### Statistical analysis

For this secondary analysis, we included women from the two studies who were in the perinatal period, defined as being pregnant or had a child of 12 months or younger [1].

First, we describe the sociodemographic characteristics, health-related status and study base of participants within the Community Study, within the Facility Study, and for the pooled sample, using percentages within each category. We created tertiles of age and education. For parity, we created three categories: antenatal, postnatal with only daughters, postnatal with any sons, which was informed by previous literature about links between gender preference and depression [16]. For disability, we created tertiles of WHODAS scores to represent those with lower (score 12–15), average (16–19) or higher (≥ 20) levels of disability.

Second, with data pooled from both studies, we evaluated whether the distribution of PHQ9 scores varied by sociodemographic characteristics, health-related status, or study base (i.e. community or facility). As the distribution of PHQ9 scores was skewed we could not use linear regression to test for associations. Instead, we report the median and interquartile range for each stratum and used the Kruskal–Wallis test to test for an association between the measure and the PHQ9 score. For the test of association between suicidal ideation and PHQ9 score, we excluded the final item on the PHQ9 from the total outcome score, as this item also pertains to suicidality. We repeated the associations analysis with a binary PHQ9 score, reported the proportion of participants in each category who screen positive (PHQ9 ≥ 10), and used Fisher’s exact to calculate P values for the differences in proportions. And then we repeated the second part of this analysis for each separate study sample.

Third, we report on the primary outcomes of the two parent studies as they pertain to this sub-sample of perinatal women. From the Community Study, we report the change, from baseline to follow-up, in the proportion of PHQ9-positive participants who reported seeking care from a health care provider for symptoms relating to depression. And from the Facility Study, we report the change from baseline to follow-up, in the proportion of PHQ9-positive participants who reported a diagnosis of depression in their clinical consultation. We tested for differences in these proportions using Fisher’s exact test.

We completed all analyses in Stata SE 14.2 (College Station, TX, USA) (Stata code in Additional file 1).

## Results

In both studies, over 99% of eligible adults who were approached by field workers opted to participate in the studies. Of the 6087 Community Study and 1577 Facility Study participants, 226 and 130 women, respectively, were in the perinatal period. The sociodemographic and health-related characteristics of women in each study are described in Table 1.

**Table 1:** Sociodemographic- and health-related characteristics of perinatal women in Sehore District, India, 2013–2016

	Community Study (n = 224)	Facility Study (n = 130)	Total (n = 354)
Age, years			
18–22	28.1	47.7	35.1
23–26	41.5	30.8	37.6
≥ 27	30.4	21.5	27.1
Education, years			
0–5	47.8	25.4	39.6
6–11	39.7	46.9	42.4
≥ 12	12.5	27.7	18.1
Religion			
Muslim	21.0	29.2	24.0
Hindu	79.0	70.8	76.0
Caste			
Scheduled caste/tribe	28.6	26.9	28.0
Other backwards caste	61.6	60.8	61.3
General/none	9.8	12.3	10.7
Housing quality			
Low	44.6	59.2	50.0
Intermediate	21.9	14.6	19.2
High	33.5	26.2	30.8
Currently pregnant			
No	68.5	20.8	50.7
Yes	31.5	79.2	49.3
Parity			
Primigravida	5.4	30.0	14.4
Daughter(s) only	32.6	24.6	29.7
≥ 1 son	62.0	45.4	55.9
Disability level			
Lower	44.6	29.2	39.0
Average	30.4	29.2	29.9
Higher	25.0	41.5	31.1
Suicidal ideation^a^			
No	93.8	94.6	94.1
Yes	6.2	5.4	5.9
Depression			
PHQ9 negative	91.1	81.5	87.6
PHQ9 positive	8.9	18.5	12.4

^a^Measured with the CIDI suicidality module [30], with 12 months recall

In the Community Study, 28.1% of women were age 18–22 years, 41.5% age 23–26 years and 30.4% age 27 or more. In the Facility Study the corresponding percentages were 47.7%, 30.8% and 21.5%, and for the pooled total sample the corresponding percentages were 35.1%, 37.6% and 27.1%. In the community, 31.5% of perinatal women were pregnant, compared to 79.2% of women recruited from the facilities. In the total sample, few—if any—women were unmarried, owned land or participated in the rural employment programme (all < 5%, data not shown). For depression screening, 8.9% of women in the community were PHQ9-positive, compared to 18.5% of women in the facilities.

The sociodemographic- and health-related factors associated with depression symptoms among participants are described in Table 2.

**Table 2:** Sociodemographic and health-related factors associated with depression symptoms among perinatal women in Sehore District, India, 2013–2016

	PHQ9 score, median (IQR)	PHQ9 ≥ 10 (%)
Total	4 (2–7)	12.4
Age, years		
18–22	*5 (3–8)*	16.8
23–26	*3 (1–6)*	8.3
≥ 27	*4 (2–7)*	12.5
Education, years		
0–5	4 (2–7)	11.4
6–11	5 (2–7)	12.7
≥ 12	4 (2–6)	14.1
Religion		
Muslim	5 (3–8)	15.3
Hindu	4 (2–7)	11.5
Caste		
Scheduled caste/tribe	4 (2–7)	10.1
Other backwards caste	4 (2–7)	13.8
General/none	4 (3–6)	10.5
Housing quality		
Low	4 (2–7)	12.4
Intermediate	4 (2–6)	7.4
High	5 (2–8)	15.6
Currently pregnant		
No	*3 (1–6)*	*8.5*
Yes	*5 (3–8)*	*16.9*
Parity		
Primigravida	5 (3–6)	11.8
Daughter(s) only	4 (2–7)	13.3
≥ 1 son	4 (2–7)	12.1
Disability level		
Lower	*2 (1–4)*	*1.4*
Average	*5 (3–7)*	*11.3*
Higher	*6 (5–10)*	*27.3*
Suicidal ideation^b^		
No	*4 (2–6)* ^a^	*10.8*
Yes	*8 (4–11)* ^a^	*38.1*
Study recruitment base		
Community	*3 (1–6)*	*8.8*
Facility	*5 (3–7)*	*18.5*

*IQR* interquartile range

Italic values indicate significance of *P* value (*P < 0.05*)

P value for difference by group calculated with Kruskal–Wallis test

P value for difference by group calculated with Fisher’s exact

^a^Using sum of PHQ9 items, excluding the suicidality item

^b^Measured using the CIDI suicidality module [30], with 12 month recall

The pooled sample of 356 women had a median PHQ9 score of 4 (IQR 2–7), and 12.4% were PHQ9 positive. Women aged 18–22 years had a median PHQ9 score of 5 (IQR 3–8) and 16.8% were PHQ9 positive. Women aged 23–26 years had a median PHQ9 score of 3 (1–6) and 8.1% were PHQ9 positive. Woman age 27 years had a median PHQ9 score of 4 (IQR 2–7), and 12.5% were PHQ9 positive. For the continuous PHQ9 score, there was evidence of a “U” shaped association with age, and positive associations with pregnancy, disability score, suicidality and being a health facility attendee (Kruskall-Wallis P < 0.05). For the binary PHQ9 score, there was evidence of positive associations with pregnancy, disability score, suicidality and being a health facility attendee (Fisher’s exact P < 0.05). The factors associated with depression symptoms for separate samples of Facility and Community Study samples are presented in Additional file 2: Table S1 and Additional file 3: Table S2.

Only 2 of 20 (10%) of PHQ9-positive women in the Community Study reported seeking treatment for their problems, and we did not find evidence of a difference between the baseline and follow up survey rounds (Fisher’s exact P > 0.05). Only 3 of 24 (12.5%) of PHQ9-positive women in the Facility Study reported a diagnosis of depression from their clinical consultation, and again we did not find evidence of a difference between baseline and follow up survey rounds (Fisher’s exact P > 0.05).

## Discussion

This is the first study to describe the distribution of perinatal depression in Madhya Pradesh, India, a predominantly (72%) rural state of 72 million people. In Sehore District, we found that 8.8% of perinatal women in the community and 18.5% of women in the facility screened positive for depression. Depression symptoms were common across different strata of sociodemographic and health-related characteristics. After implementation of a mental health care plan for integrated care in three sub-district hospitals in Sehore District [22], the proportion of screen-positive women in the community who sought care did not increase, nor was there a change in clinical detection of depression among screen-positive women in facilities.

An inverse association between socioeconomic status and perinatal depression has been demonstrated in several India-based studies [8, 10, 13, 14, 31] and in a global meta-analysis of socioeconomic status and depression [32]. As housing quality and education are proxies of family- and individual-level socioeconomic status, respectively, the absence of associations with depression symptoms was unexpected. It is possible that the effect of socioeconomic status on depression is relevant in areas with greater inequality. Sehore District is a rural area which is more uniformly lower-middle class; the difference between those with lower and higher quality housing is not as extreme as in other areas of India, and employment prospects for women are modest regardless of educational attainment. Future research can consider whether the association of socioeconomic status and depression—which is usually negative [32]—is modified by community-level inequality, or is equally valid in LMIC. And alternatively, future work can determine whether housing quality and education are adequate proxies for socioeconomic status in India.

Pressure to have a male child and disappointment over having a girl child were associated positively with depression symptoms from studies conducted in North, West and South India [7, 8, 10, 13, 14, 16, 33]. Male gender preference in Sehore District is evident from data which show the under-6 sex ratio is 927 girls for every 1000 boys [19]. It is worth noting that we did not ask directly about gender preference, and instead measured a pattern of parity. Future research should directly assess women’s gender preferences prior to giving birth, screen women for depression at the time of gender reveal (at birth, legally, in India), and should establish whether any change in depression symptoms associated with unmet gender preference is persistent.

Depression ultimately affects functioning, as evidenced by the association between PHQ9 and WHODAS measures observed here. It is the loss of functioning that makes depression a major contributor to the global burden of disease [34]. An explanatory model study in Goa, India shows that the idioms of distress among women with depression are primarily somatic, and help-seeking for these complaints was common [31]. As such, health promotion programmes which focus on functioning and somatic symptoms should consider incorporating elements of psychological counselling. For women whose disability is caused by depression, counselling directly addresses the underlying cause. For women whose disability is triggering depression symptoms, counselling confers coping skills which are likely to improve the prognosis for resolving the source of the disability.

Suicide is the second leading cause of death among young women in India [35], and in this study 5.9% of women reported suicidal ideation in the past 12 months. Commentators in India have hypothesized that suicide is not strongly linked with mental health issues in the general population [36–38]. However, limited data are available about suicide or its risk factors among perinatal women in India. Though here a statistical association was present between depression and suicidality, depression would be a poor predictor of suicide: among women who expressed suicidal ideation 72% screened negative for depression. As mental health services scale up in Madhya Pradesh, service planners must recognize that treating depression is likely to have a partial effect on reducing the level of suicidal ideation. To address the 5.9% of perinatal women who express suicidal ideation, planners must engage beyond the health sector to address determinants of suicidality which are independent of mental health problems, which as noted in a study of completed suicide among young people in South India includes factors like family conflict and domestic violence [39].

In Sehore, high levels of institutional births (81%) and antenatal tetanus vaccination (90%) [19] are evidence that health care across the perinatal period is available and accessible. Thus, using the maternal health infrastructure as a foundation for provision of maternal mental health care is worth considering. However, the quality of care is highly variable across publicly-funded health clinics in India, and inappropriate care could either trigger or exacerbate mental distress. Qualitative research can be used to establish where and from whom affected women prefer to receive depression services, and economic modelling can be used to estimate the cost–benefit associated with different service models.

Strengths of this analysis include the pooling of representative samples of perinatal women recruited from the communities and facilities of Sehore District, and use of a standardized screening tool. We must note some limitations of this study. As a secondary analysis of two parent studies, we had to use proxy measures which do not necessarily perfectly capture the underlying construct (i.e. housing quality for socioeconomic status, and parity for gender preference), and may explain the absence of associations. And as a sub-group analysis from a larger sample, our statistical power to detect weaker associations was limited by sample size, particularly for the change in care seeking and for change in clinical detection. In addition, the proportion of women who screen positive on the binary PHQ9 measure should be interpreted with caution. As with any screening tool, misclassification is likely. We mitigated against this misclassification by also analysing the PHQ9 score as a continuous measure. Even for the continuous measure, the Cronbach’s alpha of 0.67 is a potential concern, highlighting the need to adapt a Hindi-specific screening tool for depression. Yet it is worth noting the strong association of the PHQ9 with WHODAS, which is evidence of concurrent validity. Another important limitation concerns the use of the voting registration list as a sampling frame for the Community Study. A 2018 report from the Election Commission of India [40] reveals that 61% of eligible voters in Madhya Pradesh state were registered, and that factors like pregnancy, lactation and disability make women less likely to be registered. As such, we certainly underestimated the proportion of depression screen positives in the Community Study. Further, some of the associations observed here may not be generalizable to women who were not registered to vote, as they were systematically different from voters.

## Conclusions

This study highlights the key risk groups for perinatal depression—i.e. age 18–22 years, pregnant, higher disability, facility attendee—for whom mental health care should be made a priority. A recent trial demonstrated that, with training and supervision, lay health workers can deliver a manualized and adapted version of cognitive behavioural therapy to reduce depression symptoms among non-pregnant primary care attendees in India [41]. A follow up trial aims to evaluate whether peer health volunteers can do the same among pregnant women [42]; a positive result will be an impetus to promote lay counselling as a means of achieving the goals of new Mental Health Policy and Mental Healthcare Act. With adequate training and supervision, nurses and ASHA workers, who are the mainstay of national maternal and child health program in India, can be capacitated to screen vulnerable mothers and provide psychosocial interventions as indicated. In the recently published Disease Control Priorities Network, integration of mental health services into the maternal healthcare platform is recommended as being evidence-based and cost-effective [43]. Epidemiological evidence generated by this study, as well as new evidence on peer-delivered interventions for perinatal depression, must be utilized by policy-makers to prioritize mental health services for mothers along with maternal and child health services.

## Additional files

**Additional file 1.** Stata do file for analysis.

**Additional file 2: Table S1.** Sociodemographic and health-related factors associated with depression symptoms among facility-attending perinatal women in Sehore District, India, 2013–2016.

**Additional file 3: Table S2.** Sociodemographic and health-related factors associated with depression symptoms among community-based perinatal women in Sehore District, India, 2013–2016.

## Abbreviations

CIDI: Composite International Diagnostic Interview
DSM IV: Diagnostic and Statistical Manual of Mental Disorders 4th edition
LMIC: low-and middle-income countries
PHQ9: Patients Health Questionnaire 9-item
PRIME: Programme to Improve Mental Health Care
WHODAS: World Health Organisation Disability Assessment Schedule
