# Supplementary material for: Characteristics of perinatal depression in rural central, India: a cross-sectional study
Source: Int J Ment Health Syst. 2018 Nov 12;12:68. doi: 10.1186/s13033-018-0248-5 (PMC6231264; doi:10.1186/s13033-018-0248-5)
Supplement: Supplementary file 1 — Additional file 1. Stata do file for analysis. [file 13033_2018_248_MOESM1_ESM.docx]

********************************************

*** PRIME Perinatal Depression in CS/FDS ***

********************************************

local myvars submissionid round phq_5c phq? phqpos whodas_simple kids tyhos ownld hindu marit_4c age edu* sex rel_9c preg youngmonths youngyears perinatal_3c country totalphq phqpos kids suithink suiplan suiatt caste_in idate mnrega emp

use `myvars' pno chis outptcostusd suithink deptx deptx_* depdisc depdisc__friend depdisc__neighbor depdisc__spouse depdisc__parents depdisc__sib depdisc__rela depdisc__employ depdisc__other ///

using "C:\PRIME\CS\India\CS_IN.dta", clear

gen survey=1

rename chis kids_sons

append using "C:\PRIME\FDS\India\FDS_IN.dta", ///

keep(`myvars' kids_sons dx_dd ddhere)

replace survey=2 if mi(survey)

label define survey 1 Community 2 Facility

label values survey survey

label variable survey "PRIME Survey"

tab survey round, m

* Survey dates

table survey, c(n idate min idate max idate) by(round)

tab survey perinatal_3c, m

keep if perinatal_3c==2

tab survey

****************

*** NEW VARS ***

****************

xtile age_3c=age, n(3)

recode edu (99=.)(14=16)(15=18), gen(education)

label variable education "Years of education (edu)"

recode caste_in (1/2=1)(3=3)(4/5=4), gen(caste_3c)

label define caste3c 1 "SC/ST" 3 OBC 4 "General/none"

label values caste_3c caste3c

recode marit_4c (1=1)(2=2)(3/4=3), gen(marit_3c)

label define marit3c 1 Single 2 Married 3 "Post-marital"

label values marit_3c marit3c

recode kids_sons (.=0)(0=1)(1/max=2), gen(son_3c)

label define son3c 0 "No children" 1 "Only daughters" 2 "Has sons"

label values son_3c son3c

xtile whodas_3c = whodas_simple, n(3)

egen phq8total = rowtotal(phq1-phq8)

label variable phq8total "Sum of PHQ#1-PHQ#8 (excl PHQ#9 suicidality)"

sum

*************

*** ALPHA ***

*************

alpha phq1-phq9, item

sum totalphq

**************************

*** Table 1, 2, 3a, 3b ***

**************************

* Community vs facility sociodems (suicidality is for Table 1 only)

forvalues i=0/2 {

tab survey if survey!=`i'

foreach var of varlist age_3c educ_3c hindu caste_3c tyhos preg son_3c whodas_3c suithink {

tab `var' survey if survey!=`i', col

table `var' if survey!=`i', c(n totalphq p50 totalphq p25 totalphq p75 totalphq)

kwallis totalphq if survey!=`i', by(`var')

tab `var' phqpos if survey!=`i', row exact

}

* Suicidality for Table 2, 3a, 3b

foreach var of varlist suithink {

tab `var' if survey!=`i',

table `var' if survey!=`i', c(n phq8t p50 phq8t p25 phq8t p75 phq8t)

kwallis phq8total if survey!=`i', by(`var')

tab `var' phqpos if survey!=`i', row exact

}

}

* Survey design for Table 1

table survey, c(n totalphq p50 totalphq p25 totalphq p75 totalphq)

kwallis totalphq, by(survey)

tab survey phqpos, row exact

**************************

*** HELP SEEKING IN CS ***

**************************

tab phqpos round if survey==1, col

tab deptx round if phqpos==1 & survey==1, col exact

************************

*** Diagnosis in FDS ***

************************

tab phqpos round if survey==2, col

tab ddhere round if phqpos & survey==2, exact
