# Supplementary material for: Characteristics of perinatal depression in rural central, India: a cross-sectional study
Source: Int J Ment Health Syst. 2018 Nov 12;12:68. doi: 10.1186/s13033-018-0248-5 (PMC6231264; doi:10.1186/s13033-018-0248-5)
Supplement: Supplementary file 2 — Additional file 2: Table S1. Sociodemographic and health-related factors associated with depression symptoms among facility-attending perinatal women in Sehore District, India, 2013–2016. [file 13033_2018_248_MOESM2_ESM.docx]

Additional file 2: Table S1. Sociodemographic and health-related factors associated with depression symptoms among facility-attending perinatal women in Sehore District, India, 2013-2016.

|  |  | PHQ9 score, median (IQR) | PHQ9>=10 (%) |
| --- | --- | --- | --- |
| Total | | 5 (3-7) | 18.5 |
| Age, years | |  |  |
|  | 18-22 | 6 (4-8) | 22.6 |
|  | 23-26 | 5 (3-7) | 12.5 |
|  | >=27 | 5 (3-7.5) | 17.9 |
| Education, years | |  |  |
|  | 0-5 | 6 (3-7) | 18.2 |
|  | 6-11 | 5 (3-7) | 18.0 |
|  | >=12 | 5 (2.5-8) | 19.4 |
| Religion | |  |  |
|  | Muslim | 6 (4-9) | 23.7 |
|  | Hindu | 5 (3-7) | 16.3 |
| Caste | |  |  |
|  | Scheduled caste/tribe | 4 (3-7) | 11.4 |
|  | Other backwards caste | 5 (3-9) | 21.5 |
|  | General/none | 5.5 (4-8.5) | 18.7 |
| Housing quality | |  |  |
|  | Low | 5 (3-7) | 13.0 |
|  | Intermediate | 6 (3-8) | 21.0 |
|  | High | 6 (4-11) | 29.4 |
| Currently pregnant | |  |  |
|  | No | **3 (2-6)** | 11.1 |
|  | Yes | **6 (4-8)** | 20.4 |
| Parity | |  |  |
|  | Primigravida | 5 (3-6) | 7.7 |
|  | Daughter(s) only | 6 (4-8.5) | 21.9 |
|  | >=1 son | 5 (3-9) | 23.7 |
| Disability level | |  |  |
|  | Lower | **3 (2-5)** | **5.3** |
|  | Average | **5 (3-7)** | **21.0** |
|  | Higher | **6 (5-10)** | **25.9** |
| Suicidal ideation | |  |  |
|  | No | 5 (3-7)^a^ | 17.9 |
|  | Yes | 5 (3-10)^a^ | 28.6 |

**P<0.05**

IQR, Interquartile range

P value for difference by group calculated with Kruskal-Wallis test,

P value for difference by group calculated with Fisher’s exact.

^a^ Using sum of PHQ9 items, excluding the suicidality item
