# Supplementary material for: Characteristics of perinatal depression in rural central, India: a cross-sectional study
Source: Int J Ment Health Syst. 2018 Nov 12;12:68. doi: 10.1186/s13033-018-0248-5 (PMC6231264; doi:10.1186/s13033-018-0248-5)
Supplement: Supplementary file 3 — Additional file 3: Table S2. Sociodemographic and health-related factors associated with depression symptoms among community-based perinatal women in Sehore District, India, 2013–2016. [file 13033_2018_248_MOESM3_ESM.docx]

Additional file 3: Table S2. Sociodemographic and health-related factors associated with depression symptoms among community-based perinatal women in Sehore District, India, 2013-2016.

|  |  | PHQ9 score, median (IQR) | PHQ9>=10 (%) |
| --- | --- | --- | --- |
| Total | | 3 (1-6) | 8.8 |
| Age, years | |  |  |
|  | 18-22 | 4 (2-7) | 11.1 |
|  | 23-26 | 3 (1-6) | 6.3 |
|  | >=27 | 4 (2-6) | 10.3 |
| Education, years | |  |  |
|  | 0-5 | 3 (2-6) | 9.4 |
|  | 6-11 | 4 (2-7) | 9.0 |
|  | >=12 | 2 (1-5) | 6.7 |
| Religion | |  |  |
|  | Muslim | 3 (2-8) | 8.5 |
|  | Hindu | 3 (1-6) | 8.9 |
| Caste | |  |  |
|  | Scheduled caste/tribe | 3.5 (1.5-7) | 9.4 |
|  | Other backwards caste | 3 (1-6) | 9.3 |
|  | General/none | 3 (2-5) | 4.5 |
| Housing quality | |  |  |
|  | Low | 3 (1-6) | 12.0 |
|  | Intermediate | 3 (1-5) | 2.0 |
|  | High | 4 (1-7) | 9.3 |
| Currently pregnant | |  |  |
|  | No | **3 (1-5.5)** | 7.9 |
|  | Yes | **5 (2-7)** | 11.6 |
| Parity | |  |  |
|  | Primigravida | 4 (1.5-8) | 25.0 |
|  | Daughter(s) only | 3 (1-6) | 9.6 |
|  | >=1 son | 3 (1-6) | 7.1 |
| Disability level | |  |  |
|  | Lower | **2 (1-3)** | **0.0** |
|  | Average | **4 (2-6.5)** | **5.9** |
|  | Higher | **7 (5-10)** | **28.6** |
| Suicidal ideation | |  |  |
|  | No | **3 (1-6)^a^** | **6.6** |
|  | Yes | **8 (4-10)^a^** | **42.9** |

**P<0.05**

IQR, Interquartile range

P value for difference by group calculated with Kruskal-Wallis test,

P value for difference by group calculated with Fisher’s exact.

^a^ Using sum of PHQ9 items, excluding the suicidality item
